# Docosahexaenoic (DHA) modulates phospholipid-hydroperoxide glutathione peroxidase (*Gpx4*) gene expression to ensure self-protection from oxidative damage in hippocampal cells

**DOI:** 10.3389/fphys.2015.00203

**Published:** 2015-07-22

**Authors:** Verónica Casañas-Sánchez, José A. Pérez, Noemí Fabelo, David Quinto-Alemany, Mario L. Díaz

**Affiliations:** ^1^Department of Genetics, University Institute of Tropical Diseases and Public Health, University of La LagunaLa Laguna, Spain; ^2^Laboratory of Membrane Physiology and Biophysics, Department of Animal Biology, University of La LagunaLa Laguna, Spain

**Keywords:** docosahexaenoic acid, hippocampal cells, glutathione peroxidase 4, phospholipid-hydroperoxide glutathione peroxidase, transcriptional regulation, intron retention, neuroprotection

## Abstract

Docosahexaenoic acid (DHA, 22:6n-3) is a unique polyunsaturated fatty acid particularly abundant in nerve cell membrane phospholipids. DHA is a pleiotropic molecule that, not only modulates the physicochemical properties and architecture of neuronal plasma membrane, but it is also involved in multiple facets of neuronal biology, from regulation of synaptic function to neuroprotection and modulation of gene expression. As a highly unsaturated fatty acid due to the presence of six double bonds, DHA is susceptible for oxidation, especially in the highly pro-oxidant environment of brain parenchyma. We have recently reported the ability of DHA to regulate the transcriptional program controlling neuronal antioxidant defenses in a hippocampal cell line, especially the glutathione/glutaredoxin system. Within this antioxidant system, DHA was particularly efficient in triggering the upregulation of *Gpx4* gene, which encodes for the nuclear, cytosolic, and mitochondrial isoforms of phospholipid-hydroperoxide glutathione peroxidase (PH-GPx/GPx4), the main enzyme protecting cell membranes against lipid peroxidation and capable to reduce oxidized phospholipids *in situ*. We show here that this novel property of DHA is also significant in the hippocampus of wild-type mice and, to a lesser extent in APP/PS1 transgenic mice, a familial model of Alzheimer's disease. By doing this, DHA stimulates a mechanism to self-protect from oxidative damage even in the neuronal scenario of high aerobic metabolism and in the presence of elevated levels of transition metals, which inevitably favor the generation of reactive oxygen species. Noticeably, DHA also upregulated a *Gpx4* CIRT (Cytoplasmic Intron-sequence Retaining Transcripts), a novel *Gpx4* splicing variant, harboring part of the first intronic region, which according to the “*sentinel RNA hypothesis”* would expand the ability of *Gpx4* (and DHA) to provide neuronal antioxidant defense independently of conventional nuclear splicing in cellular compartments, like dendritic zones, located away from nuclear compartment. We discuss here, the crucial role of this novel transcriptional regulation triggered by DHA in the context of normal and pathological hippocampal cell.

## Introduction

Docosahexaenoic acid (DHA) is the most abundant n-3 long-chain polyunsaturated fatty acid (LCPUFA) in nerve cells. In fact, brain is the organ containing the largest amount of DHA in the whole organism, and it seems that it was selected soon in the evolution of the cephalization process of vertebrates to provide a special biochemical microenvironment to nerve cell membranes, especially during the massive accretion in the evolution of primate brains (Crawford et al., 1999, 2013; Simopoulos, 2011). DHA is a pleiotropic molecule; it is an essential component of nerve cells membranes where it esterify *sn-2* position of glycerophospholipids (mainly phosphatidylethanolamine and phosphatidylserine, the most abundant phospholipids in nerve cells) and is largely determinant of the structural and physicochemical properties of plasma membrane. Indeed, properties like membrane viscosity, lateral mobility, phase separation and microdomain segregation, conformational transitions and stability of membrane proteins, lipid-protein and protein-protein interactions, all have been shown to be modulated by DHA (Uauy et al., 2001; Stillwell and Wassal, 2003; Díaz et al., 2012, 2015). Besides its structural role, DHA participates in the modulation of neurogenesis, synaptogenesis and neurite outgrowth, refinement of synaptic connectivity, neurotransmitter release, and in memory consolidation processes (Alessandri et al., 2004; Calderon and Kim, 2004; Innis, 2007; Cao et al., 2009; Moriguchi et al., 2013), but also in the activation of signaling pathways for neuronal survival against oxidative and inflammatory cascades (Oster and Pillot, 2010; Bazinet and Layé, 2014). The importance of DHA for brain health is highlighted by the extensive epidemiological and experimental evidence linking its depletion with the development of neurodegenerative diseases (Huang, 2010; Díaz and Marín, 2013).

## The pro-oxidant environment of brain and lipid peroxidation

Chemically, DHA is a 22 carbon atoms fatty acid containing six double bonds. The presence of a double bond in the fatty acid weakens the C–H bonds on the carbon atom nearby the double bond and thus facilitates H• abstraction from a methylene group, giving rise to an unpaired electron on the carbon (–•CH–) susceptible for oxidation. This circumstance is likely to be favored in the brain parenchyma given its high metabolic rate and elevated oxygen consumption, which inevitably will produce significant amounts of reactive oxygen species as by-products, including the highly reactive superoxide anion O^−^_2_ that is converted to H_2_O_2_ (Dröge, 2002). In addition, brain is rich in redox transition metals, particularly iron, which by virtue of Fenton reaction with endogenous H_2_O_2_ produce iron(III) and generate the highly reactive hydroxyl radical OH• at the expense of endogenous reducing agents, i.e., polyunsaturated fatty acids like DHA or arachidonic acid, generating lipoperoxyl radicals. Lipid peroxidation generates hydroperoxides as well as endoperoxides, which undergo fragmentation to produce a broad range of reactive intermediates called reactive carbonyl species (RCS) such as isoprostanes (IsoPs), neuroprotanes, malondialdehyde, unsaturated aldehydes including 4-hydroxy-2-*trans*-nonenal (HNE), 4-hydroxy-2-*trans*-hexenal (HHE), and 2-propenal (acrolein), with different degrees of reactivity (Porter et al., 1995; Niki et al., 2005; Catalá, 2009; Fritz and Petersen, 2013; Naudí et al., 2013).

Clearly, the concurrency of these factors in the presence of high amounts of polyunsaturated fatty acids, mainly DHA, is expected to favor the free radical-induced peroxidation of DHA in the brain parenchyma (Van Kuijk et al., 1990; Dröge, 2002; Valko et al., 2007). An important aspect of lipid peroxidation is its self-propagating nature and fundamentally different from other forms of free radical injury in that it is a self-sustaining process capable to provoke extensive brain tissue damage (Catalá, 2009; Singh et al., 2010). As most lipids in the brain are contained in the membrane phospholipids, the main outcome of lipid peroxidation is the structural damage of membranes, causing structural changes that impact membrane fluidity and permeability, neurotransmission, signaling, ion transport, and impaired electrical conduction.

Nerve cells are endowed with different antioxidant systems that render them protected from oxidative damage caused by lipid peroxides. This protection is mainly accomplished by phase II detoxifying enzymes belonging to two antioxidant systems, namely thioredoxin and glutathione systems, which use hydrophilic thiol-containing molecules (thioredoxin and glutathione, respectively), as electron donors to generate conjugated metabolites (Arnér and Holmgren, 2000; Imai and Nakagawa, 2003). However, although members of the thioredoxin system (mammalian thioredoxin reductases) are capable to reduce some non-disulfide-containing molecules, including lipid hydroperoxides independently of thioredoxin (Björnstedt et al., 1995), it is the only within the glutathione system where enzymes exist that are capable to recover oxidized membrane lipids (Imai and Nakagawa, 2003).

## Phospholipid-hydroperoxide glutathione peroxidase and membrane protection

Phospholipid hydroperoxide glutathione peroxidase, glutathione peroxidase 4, or GPx4, is a member of glutathione peroxidases family of selenoproteins, most of which bear a selenocystein as catalytically active amino acid, which confers a more efficient reaction with peroxide substrates (Nauser et al., 2006). GPx4 uses preferentially glutathione as electron donors as long as cellular concentrations of the reduced form (GSH) are not limiting, but may accept other thiol groups in proteins as reducing equivalent (Godeas et al., 1997). Therefore, GPx4 can either act as a GSH peroxidase or a thiol peroxidase depending on the availability of GSH (Brigelius-Flohé and Maiorino, 2013). However, unlike other glutathione peroxidases, GPx4 is capable of reducing complex lipid peroxides, like phospholipid hydroperoxides, even when integrated in highly structured lipid-protein assemblies such as lipoproteins and membranes (Imai and Nakagawa, 2003). X-ray data indicated that, in contrast to other GPx isoforms, the active site of GPx4 lacks a surface-exposed loop domain which appears to limit the accessibility of large oxidized substrates, but instead contains a large hydrophobic surface that allows GPx4 to closely associate with membranes and lipoproteins (Scheerer et al., 2007). Its ability to directly reduce phospholipid hydroperoxides in membranes without prior action of phospholipase A2 membrane makes GPx4 unique amongst antioxidative enzymes (Imai et al., 2003; Savaskan et al., 2007). Further, unlike other glutathione peroxidases (GPx1, GPx2, GPx3, GPx5, and GPx6) which exist as homo(tetra)-oligomeric proteins, GPx4 is a monomeric protein of about 20 kDa and misses the tetramer interfaces for oligomer formation (Scheerer et al., 2007; Brigelius-Flohé and Maiorino, 2013).

GPx4 is also peculiar amongst glutathione peroxidases because of its additional regulatory functions. Thus, GPx4 is considered not only a phase II antioxidant enzyme but is also endowed with functions associated with the regulation of apoptosis, gene expression, eicosanoid biosynthesis, and embryo development (Imai and Nakagawa, 2003; Savaskan et al., 2007; Ufer and Wang, 2011). The essential nature of GPX4 is demonstrated by the fact that genetic disruption of the entire *Gpx4* gene in homozygous *Gpx4* knockout causes *in utero* fetal death by midgestation (Imai et al., 2003; Yant et al., 2003). This intrauterine lethality has been related to increased apoptosis and cell death leading to malformation of embryonic structures and major defects in brain development (Imai et al., 2003; Yant et al., 2003). This contrasts profoundly with the knockouts on *Gpx1–3* genes, which are viable though more sensitive to stressors (Brigelius-Flohé and Maiorino, 2013), indicating a degree of redundancy of these genes for at least some of their biochemical functions, i.e., oxidant scavengers, *in vivo*.

**Figure 1:**
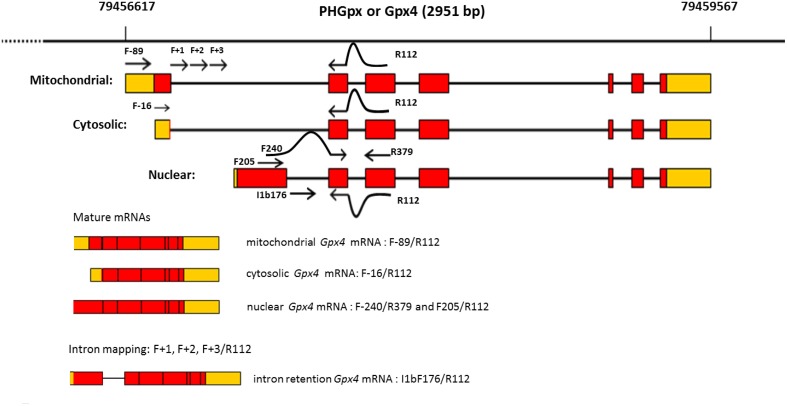
**Structural organization of mouse Gpx4 gene**. Boxes indicate coding exons (red) and UTRs (orange). Bold lines represent intronic sequences. Sites targeted by amplification primers are shown as arrowed lines.

In mammalian cells GPx4 is found in the cytoplasm, nucleus, mitochondria, and also in the endoplasmic reticulum (Imai and Nakagawa, 2003). From the molecular point of view, GPX4 is also singular because the three isoenzymes, namely cytosolic GPx4 (c-GPx4), mitochondrial GPx4 (m-GPx4), and nuclear GPx4 (n-GPx4), all derive from a single gene (Figure 1), but that can be distinguished by their N-terminal sequences (Imai and Nakagawa, 2003; Savaskan et al., 2007). The *Gpx4* gene comprises 7 exons, of which exons 2–7 encoding for the functional enzyme are shared by the three isoforms. The differential N-terminal sequences are attained by three major start sites for translation (Figure 1) that reside in two alternative exons 1 (E1a and E1b). Thus, exon 1a contains two in-frame translational start sites (5′AUG and 3′AUG) separated by a sequence that encode for a mitochondrial leader peptide. Translation initiation at the 5′AUG results in the generation of m-GPx4 isoenzyme, while translation from the 3′AUG, that lacks this leader signal, yields c-GPx4. Because the mitochondrial leader peptide is cleaved off after import into mitochondria, c-GPx4 and m-GPx4 cannot be differentiated on the basis of their primary structure. Finally, the alternative first exon (E1b) encodes the N-terminal part of the nuclear isoform, n-GPx4, and contains a nuclear targeting sequence which is apparently retained after nuclear import (Pfeifer et al., 2001), and makes this isoform distinguishable from m/c-GPx4 at protein level.

## Unexpected DHA-induced transcriptional regulation of *Gpx4* gene

We have recently reported that DHA upregulates several members of both glutathione/glutaredoxin and thioredoxin/peroxiredoxin antioxidant systems in mouse hippocampal HT22 cells (Casañas-Sánchez et al., 2014). Noticeably, within the glutathione/glutaredoxin system, largest changes in gene expression were observed for the *Gpx4* isoforms, around 150%, and affecting all transcripts analyzed, including the mitochondrial, cytosolic, and nuclear isoforms. The change induced by DHA on glutathione peroxidase genes was specific for *Gpx4* since no variation was observed for the cytosolic *Gpx1* gene (Casañas-Sánchez et al., 2014). Paralleling these changes, DHA treatment significantly increased GPx4 (and total GPx) activities, following a time-course that was compatible with the necessary delay for *Gpx4* mRNA translation. Importantly, these effects of DHA were specific for DHA and were not be detected when DHA was replaced by arachidonic acid, another highly abundant polyunsaturated fatty acid in nerve cells, under identical experimental conditions (Casañas-Sánchez et al., 2014).

**Figure 2:**
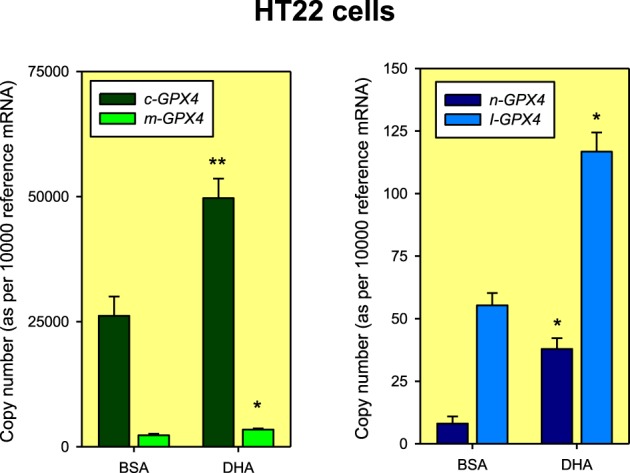
**Absolute quantification of *Gpx4* mRNA isoforms in HT22 cells exposed to BSA (Vehicle: Bovine serum albumin) or DHA for 48 h**. The normalization factor (reference mRNA) for each cDNA sample was calculated as the geometric mean of the expression values of reference genes *Hprt1* and *Tbp* genes using standard curves generated from purified amplicons as described in Expósito-Rodríguez et al. (2011). Data are expressed as mean ± SEM from four different experiments. Statistical comparisons were assessed using Mann-Whitney *U*-test for independent samples. ^*^p < 0.05 and ^**^p < 0.01 compared to BSA.

Absolute quantification of *Gpx4* isoform expression in unstimulated HT22 cells revealed that the largest expression values corresponded to the cytoplasmic variant and the lowest for the nuclear mRNA variant, with a difference of about 3 orders of magnitude (Figure 2). Furthermore, in absolute terms, we observed that DHA treatment increases nearly 1.5-fold the expression of m/c-*Gpx4*, which must be attributable to the enhancement of the mRNA coding for the cytoplasmic isoform, whose abundance is about one order of magnitude higher than for m-*Gpx4*. The nuclear isoform was also up-regulated by DHA, but its absolute magnitude was negligible compared to m/c-*Gpx4* (Figure 2).

**Figure 3:**
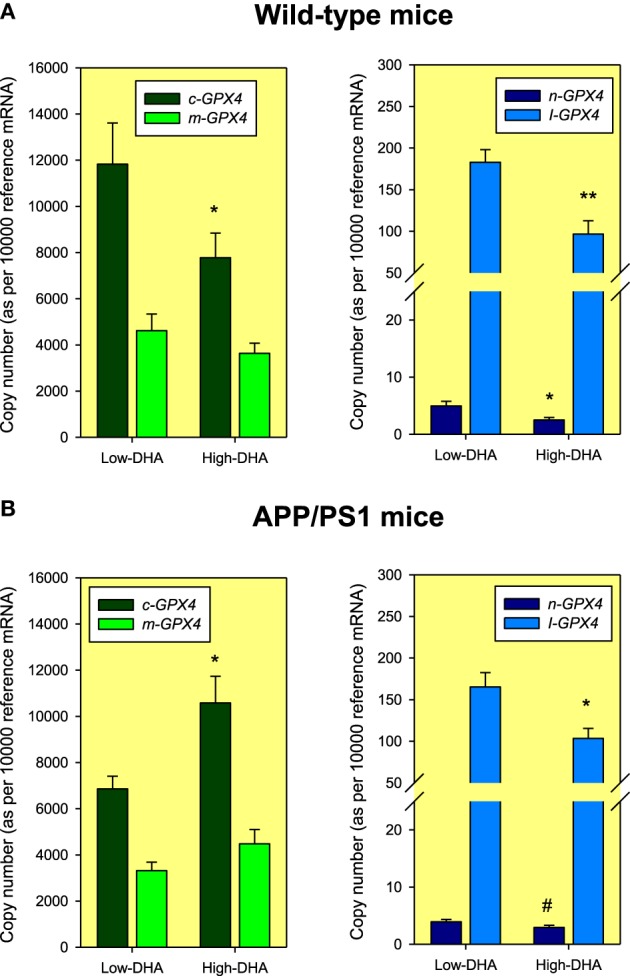
**Absolute quantification of *Gpx4* mRNA isoforms in 6 months old Wild-type (A) and APP/PS1 (B) mice fed Low-DHA or High-DHA diets for 3 months**. Data are expressed as mean ± SEM from four different animals under each dietary condition and genotype. The normalization factor (reference mRNA) for each cDNA sample was calculated as the geometric mean of the expression values of reference genes *Hprt1* and *Tbp* genes using standard curves generated from purified amplicons as described in Expósito-Rodríguez et al. (2011). Statistical comparisons were assessed using Mann-Whitney *U*-test for independent samples. ^*^p < 0.05, ^**^p < 0.01, and ^#^*p* < 0.1 compared to Low-DHA.

Using identical amplicons (Table 1), we further explored for the presence of these different *Gpx4* isoforms in the hippocampus of C57BL/6 mice. The results revealed the presence of all different isoforms, and noticeably, following a similar expression pattern, with m/c-*Gpx4* being the most abundant transcripts and n-*Gpx4* showing lowest expression. Interestingly, animals exposed to High or Low-DHA diets also exhibited differential *Gpx4* transcriptional regulation in response to DHA-containing or DHA-impoverished diets and differentially affected between wild-type and APP/PS1, a familial model of Alzheimer's disease (Figure 3). Thus, Absolute quantification of *Gpx4* isoforms in WT animals, revealed that Low-DHA diets leads to the stimulation of gene expression of all isoforms (between 1.43 and 1.99 times), being the largest stimulation observed for the cytosolic isoform, which, in turn is the most abundant *Gpx4* isoform under any circumstance (Figure 3). These observations strongly suggest a compensatory genetic strategy aimed to ensure protection of membrane DHA from oxidative damage under conditions of limited DHA availability. Overall these data indicates that the lack of sufficient DHA or its deficient supply is accompanied by increased *Gpx4* mRNAs expression and GPx4 protein synthesis, which consequently augment cellular resistance to oxidative damage of DHA-containing phospholipids. A different behavior was observed in APP/PS1 animals, where the high-DHA containing diet stimulated the expression of cytoplasmic and mitochondrial *Gpx4* mRNAs. The different expression levels observed in APP/PS1 animals, especially for the m-*Gpx4* isoform, suggest a genotype-related transcriptional regulation, which fits well with the increased levels of hippocampal oxidative stress demonstrated in transgenic animals (Aso et al., 2012) and also the lower levels of hippocampal DHA compared to WT animals (Fabelo et al., 2012).

**Table 1:** **Oligonucleotides used as primers for quantification of different *Gpx4* mRNA isoforms and for mapping 5′ end of *I*-*Gpx4* mRNA in HT22 cells and mouse hippocampus**.

**Targeted gene**	**Primers**	**Exon/intron**	**Amplicon size (bp)**	**gDNA discrimination**
*m-Gpx4*	F-89: CCgCCgAgATgAgCTgg	E1a	128	No
	R112: TgCACACgAAACCCCTgTACT	E2-E3		
*m/c-Gpx4*	F-16: TggTCTggCAggCACCAT	E1a	201	No
	R112: TgCACACgAAACCCCTgTACT	E2-E3		
*n-Gpx4*	F240: gTTCCTgggCTTgTgTgCAT	E1b-E2	140	No
	R379: AggCCACgTTggTgACgAT	E3		
*n/I-Gpx4*	F205: CTgCAAgAgCCTCCCCAgT	E1b	157/370	Yes
	R112: TgCACACgAAACCCCTgTACT	E2-E3		
*I-Gpx4*	FI1b176: ggACCTgggTTAggACACTCA	I1b	147	Yes
	R112: TgCACACgAAACCCCTgTACT	E2-E3		
Mapping (+1)	F+1: gTgggCTACTggGAACTTgg	I1a	907	
	R112: TgCACACgAAACCCCTgTACT	E2-E3		
Mapping (+2)	F+2: gggAAAgCggAgCCTgATAg	I1a	787	
	R112:TgCACACgAAACCCCTgTACT	E2-E3		
Mapping (+3)	F+3: CTTggCTACCggCTCTTTg	I1a	630	
	R112: TgCACACgAAACCCCTgTACT	E2-E3		
*Hprt1*	F: TCAgACTgAAgAgCTACTgTAATgA	E3-E4	136	Yes
	R: AAgTTTgCATTgTTTTACCAgTg	E6		
*Tbp*	F: gACCCACCAgCAgTTCAgTAg	E6	136	Yes
	R: CTCTgCTCTAACTTTAgCACCTgT	E7-E8		

*Determination of absolute mRNA levels by real-time quantitative PCR (RT-qPCR) were performed as described in Expósito-Rodríguez et al. (2011). Hprt1, Hypoxanthine guanine phosphoribosyl transferase 1; Tbp, TATA binding protein*.

Finally, using appropriate amplicons (Table 2) we tested for the presence and absolute abundance of the different *GPX4* transcripts in the human cell line SHSY-5Y. The results revealed that all *GPX4* isoforms are expressed in this cell line and, more interestingly, that their absolute abundance followed the same sequence observed in HT22 cells, though in this case the amount of the most prominent isoform, namely c-*GPX4*, was notably higher (about four orders of magnitude) than any other isoform.

**Table 2:** **Oligonucleotides used as primers for quantification of different *GPX4* mRNA isoforms and for mapping 5′ end of I-*GPX4* mRNA in SHSY-5Y cells**.

**Targeted gene**	**Primers**	**Exon/intron**	**Amplicon Size (bp)**	**gDNA discrimination**
*m-GPX4*	F-E1m: CATTggTCggCTggACgAg	E1a	242	Yes
	R-E34: CACACgAAgCCCCggTACT	E2-E3		
*m/c-GPX4*	F-E1c: CCTggCCgggACCATg	E1a	123	Yes
	R-E34: CACACgAAgCCCCggTACT	E2-E3		
*n- GPX4*	F-E2si: CAggCAgCggTgCCAgAg	E1b	170	Yes
	R-E23: gggACgCgCACgggTC	E1b-E2		
*n/I- GPX4*	F-E2_*N*_: gATCCACgAATgTCCCAAgTC	E1b	138/394	Yes
	R-E34: CACACgAAgCCCCggTACT	E2-E3		
*I- GPX4*	F-I1b: gAggAgCgTTCAggTCTTCAg	I1b	242	No
	R-E34: CACACgAAgCCCCggTACT	E2-E3		
Mapping (+1)	F+1: gTgAgCTAgCgCCgCg	I1a	1165	
	R-E34: CACACgAAgCCCCggTACT	E2-E3		
Mapping (+2)	F+2: CCCTCCAggCCgTTgTAgg	I1a	978	
	R-E34: CACACgAAgCCCCggTACT	E2-E3		
Mapping (+3)	F+3: CggAgggCTggAAATCCC	I1a	730	
	R-E34: CACACgAAgCCCCggTACT	E2-E3		
*HPRT1*	F: gACCAgTCAACAggggACAT		173	Yes
	R:AACACTTCgTggggTCCTTTTC			
*RPL32*	F: CATCTCCTTCTCggCATCA		153	Yes
	R:AACCCTgTTgTCAATgCCTC			

*Determination of absolute mRNA levels by real-time quantitative PCR (RT-qPCR) were performed as described in Expósito-Rodríguez et al. (2011). HPRT1, Hypoxanthine guanine phosphoribosyl transferase 1; RPL32, Ribosomal protein L32*.

## Defining a unified rationale for DHA-induced regulation of *Gpx4* gene expression

**Figure 4:**
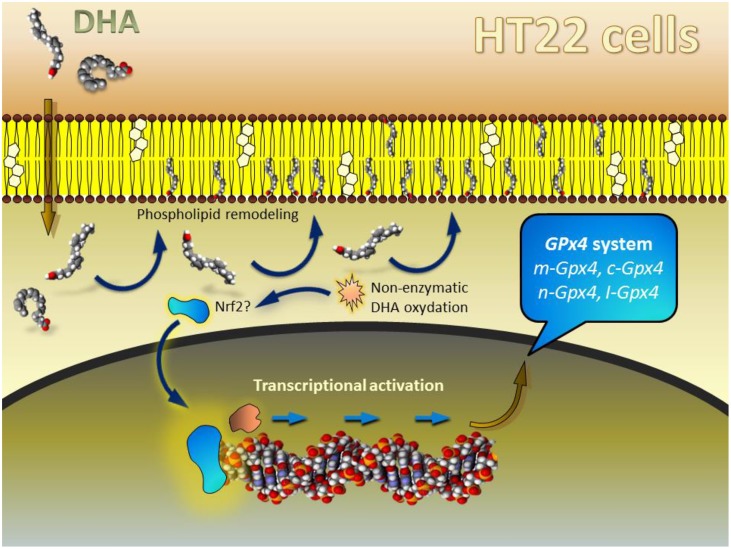
**Schematic model depicting the hypothetical mechanism of DHA-mediated regulation of *Gpx4* in HT22 cells**. For details see “*Defining a unified rationale for DHA-induced regulation of Gpx4 gene expression*.” Modified from Casañas-Sánchez et al. (2014).

Our initial observations in HT22 indicated that the upregulation of *Gpx4* expression occurred with a significant delay form the initial exposure to DHA. Thus, it was necessary to expose cells for 48 h to detect the significant change in the expression levels of all *Gpx4* isoforms (Casañas-Sánchez et al., 2014). Our interpretation of these findings was related to the fact that control unstimulated HT22 cells (as most neuronal cell lines studied so far) contain extremely low levels of DHA in their membrane phospholipids (Martín et al., 2006). Supplementation of culture medium with DHA causes a nearly immediate incorporation by deacylation-reacylation mechanisms, whereby some monounsaturated fatty acids (mainly oleic acid) are readily replaced with DHA in membrane phospholipids (Farooqui et al., 2000; Martín et al., 2006). The activation of the transcriptional process occurred only after membrane phospholipids were replenished, because only then enough DHA remained unesterified in the cytoplasm of neuronal cells, being then susceptible for non-enzymatic oxidation in the prooxidant cellular environment (Figure 4). Indeed we could only detect significant levels of the specific DHA-derived lipoperoxide 4-hydroxy-2-hexenal (HHE) after 36 h exposure to DHA, just before upregulation of *Gpx4* expression occurred. These findings were interpreted as HHE providing the signal to trigger transcriptional regulation, likely through activation of Nrf2 transcription factor. Indeed, it is known that Nrf2 is a master factor for the regulation of antioxidant response elements (ARE) in several antioxidant and phase II detoxifying proteins in different cell types, including nerve cells (Kobayashi and Yamamoto, 2005; Zhang et al., 2013), and several reports have shown that although DHA itself does not bind Nrf2, it may stimulate transcriptional activity of ARE-containing genes through activation of Nrf2 upon generation of 4-hydroxy-2-hexenal, which indeed activates Nrf2 (Ishikado et al., 2013; Kusunoki et al., 2013; Yang et al., 2013).

The results obtained in the hippocampus of WT mice were particularly interesting, since transcriptional activation of all *Gpx4* isoforms was significantly higher under the Low-DHA dietary condition compared to the High-DHA diet. Though apparently these results might contradict the results obtained in HT22 cells, the outcomes suggest that it is under a low DHA diet with limited availability of n-3 precursors, when it is especially important to activate mechanisms (like upregulation of *Gpx4* isoform expression) that may ensure the preservation of DHA in brain membranes. In fact, analyses of hippocampal lipid composition in WT and APP/PS1 animals revealed a significant reduction of DHA contents in Low-DHA diet compared to the High-DHA diet, yet the amount of DHA in the Low-DHA condition was surprisingly higher than expected from the dietary supply (Table 3), which reflects an extremely efficient ability to preserve brain DHA even when subjected to negligible levels in the diet (Díaz et al., accepted). This exceptional capability is mainly attributable to the favorable gradient for fatty acids to cross the blood-brain barrier, either passively or via fatty acid binding proteins (FATPs), since once in the brain, unesterified fatty acids are converted to CoA thioesters by the long-chain-fatty-acid-CoA synthase (ACSL) family of proteins, therefore maintaining a continuous concentration gradient (see (Bazinet and Layé, 2014) for comprehensive review).

**Table 3:** **Fatty acid composition of total lipids in the hippocampus of 6 months old WT and APP/PS1 mice, and fed for 3 months with either Low-DHA or High-DHA diets**.

**Fatty acid**	**WT**		**APP/PS1**	
	**Low-DHA**	**High-DHA**	**Low-DHA**	**High-DHA**
14: 0	0.10 ± 0.00^*^^#^	0.12 ± 0.00^#^	0.12 ± 0.01	0.13 ± 0.00
16: 0 DMA	2.16 ± 0.01	2.12 ± 0.01	2.11 ± 0.04	2.13 ± 0.04
16: 0	18.41 ± 0.06^#^	18.58 ± 0.16	17.80 ± 0.16	18.25 ± 0.20
18: 0 DMA	4.07 ± 0.04	4.09 ± 0.06	4.01 ± 0.15	4.04 ± 0.05
18:1 n-9 DMA	1.51 ± 0.04	1.54 ± 0.03	1.52 ± 0.08	1.52 ± 0.06
18:1 n-7 DMA	1.94 ± 0.05	2.01 ± 0.04	2.09 ± 0.05	1.99 ± 0.08
18: 0	19.60 ± 0.10^*^	19.05 ± 0.01	19.54 ± 0.12	19.14 ± 0.30
18: 1 n-9	15.81 ± 0.06^*^^#^	17.04 ± 0.11	16.32 ± 0.16	17.00 ± 0.25
18: 1 n-7	3.59 ± 0.06^*^	3.40 ± 0.01	4.18 ± 0.60	4.11 ± 0.56
18: 2 n-6	0.49 ± 0.01	0.48 ± 0.02	0.50 ± 0.02	0.48 ± 0.03
20: 0	0.28 ± 0.01	0.26 ± 0.01	0.29 ± 0.01	0.26 ± 0.02
20: 1 n-9	1.76 ± 0.05	1.80 ± 0.03	1.96 ± 0.07	1.74 ± 0.11
20: 1 n-7	0.34 ± 0.01	0.36 ± 0.00	0.37 ± 0.02	0.36 ± 0.02
20: 3 n-6	0.28 ± 0.01^*^	0.57 ± 0.00^#^	0.28 ± 0.01^*^	0.54 ± 0.01
21: 0	0.02 ± 0.01	0.00 ± 0.00	0.00 ± 0.00	0.00 ± 0.00
20: 4 n-6	9.87 ± 0.16^*^^#^	7.64 ± 0.02	9.34 ± 0.07^*^	7.54 ± 0.19
20:5 n-3	0.00 ± 0.00^*^	0.19 ± 0.01	0.00 ± 0.00^*^	0.21 ± 0.01
22:0	0.22 ± 0.01	0.21 ± 0.00	0.24 ± 0.01	0.21 ± 0.02
22: 4 n-6	3.05 ± 0.06^*^	1.85 ± 0.02	3.14 ± 0.10^*^	1.82 ± 0.07
22: 5 n-6	1.19 ± 0.02^*^^#^	0.09 ± 0.00	0.87 ± 0.04^*^	0.09 ± 0.01
22: 5 n-3	0.03 ± 0.01^*^	0.39 ± 0.01	0.01 ± 0.01^*^	0.39 ± 0.01
24: 0	0.26 ± 0.01	0.26 ± 0.01	0.29 ± 0.02	0.27 ± 0.03
22: 6 n-3	12.69 ± 0.21^*^^#^	16.66 ± 0.10 Δ	11.19 ± 0.11^*^	15.04 ± 0.55
24: 1 n-9	0.85 ± 0.03^#^	0.83 ± 0.04^#^	1.19 ± 0.09^*^	0.70 ± 0.01
**TOTALS AND INDEXES**				
Saturates	45.35 ± 0.05^*^	44.84 ± 0.12	44.61 ± 0.38	44.59 ± 0.49
Unsaturated	54.54 ± 0.05^*^	55.05 ± 0.12	55.27 ± 0.39	55.28 ± 0.50
DMAs	9.67 ± 0.08	9.76 ± 0.12	9.74 ± 0.31	9.68 ± 0.15
n-9	20.34 ± 0.18^*^^#^	21.62 ± 0.16	21.53 ± 0.15	21.43 ± 0.56
n-3	12.80 ± 0.21^*^	16.31 ± 0.09	11.27 ± 0.12^*^	15.16 ± 0.55
n-6	14.99 ± 0.22^*^^#^	10.70 ± 0.04	14.23 ± 0.12^*^	10.53 ± 0.27
n-3/n-6	0.85 ± 0.02^*^	1.52 ± 0.01	0.86 ± 0.01^*^	1.53 ± 0.03
18:1 n-9/n-3 H	1.24 ± 0.02^*^^#^	1.06 ± 0.01	1.34 ± 0.02^*^	1.07 ± 0.06
Unsaturation Index	163.12 ± 0.99^*^^#^	166.48 ± 0.30	158.64 ± 0.53	165.50 ± 2.94
saturates/n-3	3.55 ± 0.05^*^	2.75 ± 0.01	3.64 ± 0.03^*^	2.77 ± 0.07
saturates/n-9	2.23 ± 0.02^*^^#^	2.07 ± 0.02	2.07 ± 0.02	2.09 ± 0.07

Results are expressed as mole % and represent means ± SEM of four different animals under each dietary condition and genotype. DMA: Dimethyl acetals.

* *p < 0.05 compared to High-DHA*.

# *, Δ : p < 0.05 and p < 0.1 compared to APP/PS1. Fatty acid extraction and determination was performed as described in Fabelo et al. (2012) and Martín et al. (2006). Data were analysed by One-Way ANOVA followed by Tukey's multiple comparison test. Comparisons between Low-DHA and High-DHA treatments were performed using Student's t-test or Mann-Whitney U-test where appropriate. Main monoenoic (oleic acid, 18:1n9), n-6 LCPUFA (arachidonic acid, 20:4n-6), and n-3 LCPUFA (DHA, 22:6n-3) are highlighted on pink background*.

As mentioned before, APP/PS1 animals also responded to the different diets with changes in the transcriptional levels of *Gpx4* expression. However, we only observed increased expression of c-*Gpx4* isoform, and in the High-DHA diet. The apparent discrepancy between WT and APP/PS1 animals is likely related to the involvement of constitutive oxidative stress associated to amyloid generation in the brain of transgenic animals (Aso et al., 2012; Fabelo et al., 2012). Indeed, it is now widely accepted that amyloid peptide induces oxidative stress, and inflammation and oxidative stress generate more Aβ, giving rise to a vicious cycle between Aβ and free radical formation/lipid peroxidation in the presence of transition metals (Behl et al., 1994; Pamplona et al., 2005; Solfrizzi et al., 2006). In line with this matter, increased levels of LCPUFA–derived lipid hydroperoxides like HHE, HNE, and other endoperoxides like isoprostanes have been observed in the brain of transgenic models of Alzheimer's disease, including 3xTgAD and APP/PS1 animals, and more relevantly, in different areas of human brain and cerebrospinal fluid of AD patients (Marcus et al., 1998; Markesbery and Lovell, 1998; Praticò et al., 1998; Valko et al., 2007; Gwon et al., 2012). In this order of ideas, it seems plausible that in the high-DHA diet, lipid-derived hydroperoxides must be elevated in the more prooxidant environment of brain parenchyma in transgenic animals, including DHA-related HHE, which may trigger the stimulation of Nrf2 transcription factor, which eventually would favor the activation of a transcriptional program to increase *Gpx4* isoforms expression, especially the cytoplasmic isoform. In agreement with our findings, recent genomic gene expression profiles by microarray analyses in a “gene dose-response” model of Nrf2-null model have revealed that graded activation of Nrf2 increased the transcription of a large number of genes involved in Nrf2-mediated oxidative stress response, glutathione metabolism, and xenobiotic metabolism, including several genes responsible for the reduction of superoxide ions using GSH, i.e., glutathione peroxidases *Gpx2* and *Gpx4*, which augmented by 2260 and 105%, respectively, in Keap1-HKO mice compared to WT mice (Wu et al., 2011).

Alternatively, altered genetic regulation of *Gpx4* expression caused by overexpression of mutated APP and PS1 in transgenic animals, may have disturbed the normal gene expression pattern, as it has been recently demonstrated to occur in the frontal cortex and hippocampus of transgenic mice carrying the Δ E9 mutant hPS1 gene (Unger et al., 2005). Likewise, recent comparative studies in sporadic (icv-STZ Mouse) and familial (3xTg-AD mouse) mice models of AD have revealed the transcriptional alteration of more than 80 genes related to synapse function, apoptosis and autophagy, AD-related protein kinases, glucose metabolism, insulin signaling, and mTOR pathway in the hippocampus and the cerebral cortex, not only when compared to WT animals, but also between both AD models (Chen et al., 2012).

In summary, we hypothesize that by upregulating *Gpx4* gene expression and increasing GPx4 protein levels and enzyme activity, DHA initiates a self-protective biochemical strategy in the hippocampal neuron to control non-enzymatic peroxidation of DHA-containing phospholipids in the membrane from cellular and subcellular compartments. Given the extremely low capacity of hippocampal cells to biosynthesize DHA (reviewed in Plourde and Cunnane, 2007; Bazinet and Layé, 2014), and the essential nature of this fatty acid in the nervous system, this strategy provides an adaptive mechanism to keep the high DHA levels in hippocampal phospholipids.

## Retained intronic sequences in *Gpx4* mRNA

In initial experimental strategy used for the detection of the different *Gpx4* mRNAs, we designed a couple of primers targeted to exons E1b (forward primer) and the boundary between exons E2/E3 (reverse primer) for the specific detection of nuclear *Gpx4* mRNA. Unexpectedly, in these experiments we detected the presence of two amplicons: one of the expected molecular size (157 bp) for a fully processed n-*Gpx4* mRNA, and another fragment 370 bp. Once sequenced, we observed that this later amplicon contained an extra 213 bp sequence corresponding to intron I1b located between exons E1b and E2. This unexpected sequence (named here I-*Gpx4*) was detected in control HT22 cells (treated with vehicle), but more interestingly, that its expression level was subjected to significant upregulation by DHA treatment (near 100% compared to control cells), which was in the range observed for the changes in expression for the set of other *Gpx4* mRNA variants (Figure 2). In absolute terms, I-*Gpx4* quantification revealed that this isoform was nearly 3 times more abundant than n-*Gpx4* under unstimulated conditions, and about 7 times higher after DHA treatment, pointing to a significant biological role of this intron-retaining isoform in hippocampal cells. Nonetheless, I-*Gpx4* was expressed a much lower amount than c-*Gpx4*, even after DHA treatment.

In order to gain a deeper insight on the transcription start site for the intron-retained transcript, we designed several amplification primers directed to intron I1a (Figure 1 and Table 1). While the primers targeted to E1b exon and intron I1b amplified the expected amplicons, all other primers targeting E1b exon upstream sequences were unsuccessful in these PCR assays. These results strongly indicate that a *Gpx4* CIRT transcript represents an unprocessed variant of the n-*Gpx4* mRNA isoform.

In order to assess for the biological relevance of this intron-retaining *Gpx4* isoform, we used these specific amplicons to unveil the presence in the hippocampus of C57Bl/6 mice. We observed that not only the amplicon was detected, but also that its expression was modulated by the contents of DHA in the diet. Thus, levels of I-*Gpx4* isoform were significantly higher (around 2 times) in animals receiving Low-DHA diet compared to animals receiving a High-DHA diet. A similar result was obtained in transgenic APP/PS1 animals, though in this case the increase in I-*Gpx4* gene expression was around 1.6 times higher in animals maintained in a Low-DHA diet (Figure 3). Absolute comparisons between WT and APP/PS1 animals revealed that I-*Gpx4* was higher in WT animals than in the APP/PS1 genotype. Overall these data indicates that the lack of sufficient DHA or its deficient supply is accompanied by an increase in I-*Gpx4*. Again, these higher expression levels observed in APP/PS1 animals suggests a genotype-related transcriptional regulation, which may be related to increased hippocampal oxidative stress in transgenic animals (Aso et al., 2012).

Whether this regulated *Gpx4* mRNA variant constitutes an intermediate processing stage of *Gpx4* splicing or represents a different level of transcriptional regulation by intron retention remains unrevealed. However, the notion of intron retention has been gaining consistency in the last decade, as an alternative processing of conventional (nuclear) mRNA, adding a novel level of complexity in gene expression regulation. These sequences, named as CIRTs (Cytoplasmic Intron-sequence Retaining Transcripts), comprise only few introns and are limited to small fractions of introns within the gene (Buckley et al., 2014). Indeed, in recent years, a number of transcripts containing intronic sequences and subjected to cytoplasmic splicing have been unveiled across a number of cell types and species (Buckley et al., 2014), and the list keeps growing steadily. Noticeably, a recent study performed in mouse brains have revealed the broad presence of cytoplasmic intron-sequence retaining transcripts in hippocampal neurons, with 59.4% of total gene transcripts in dendrites containing intronic reads (Khaladkar et al., 2013). Examples of retained introns from individual transcripts of relevance in neuronal cells include the large-conductance calcium-dependent potassium channel (BK) encoded by *KCNMA1* gene (Bell et al., 2008, 2010), γ-subunit of GABA-A receptor (encoded by *GABRG3*) (Buckley et al., 2011), ionotropic Glutamate receptor (encoded by *GRIK1* gene) and type 1 NMDA receptor (encoded by GRIN1) (Buckley et al., 2011) or the α-1 subunit of T type, voltage-dependent Calcium channel (encoded by *CACNA1H*) (Zhong et al., 2006). Though the biological relevance of CIRTs is still largely unknown, their abundance in neuronal dendrites (and also in enucleated platelets) suggest that endogenous cytoplasmic transcripts harboring retained intronic substrates could be subject to extranuclear splicing, thereby obviating the need for shuttling molecules to and from the nucleus for processing (Buckley et al., 2014). In this manner, dendritic splicing could represent a novel method of post-transcriptional gene regulation.

In this order of ideas, it is tempting to speculate that CIRT in *Gpx4-I* gene may be part of the normal endogenous post-transcriptional regulation pathway. Such regulated extranuclear splicing variants may be part of what Buckley et al. (2014) designed as “sentinel RNA hypothesis,” according to which a master transcript harboring CIRTs would serve to generate transcript variants within the cytoplasm in response to specific stimulation, or under conditions in which a rapid processing is required and adaptive. A plausible scenario for this stimulus-dependent cytoplasmic splicing of I-*Gpx4* in hippocampal cells, is that this transcript processing in cellular domains distant from cellular nucleus, as is the case of dendrites, would enhance the antioxidant buffering capacity against the acute generation of lipid peroxides by oxidative insults, which would commit the integrity of membrane unsaturated phospholipids, especially those enriched in DHA.

## Conclusions and hypothesis

In summary, current available data indicate that *Gpx4* gene is subjected to a complex transcriptional regulation that renders nuclear, cytoplasmic, and mitochondrial isoforms, but also generate an intron-retaining variant, likely corresponding to a CIRT, all derived from the same gene. At least in hippocampal HT22 cells and in the hippocampus of wild type mice, the transcriptional control of most of these *Gpx4* isoforms is modulated by DHA following an unprecedented homeostatic strategy aimed to buffer its potential oxidation and to ensure its preservation in the hippocampal neuronal membrane. The observations in transgenic APP/PS1 mice, suggest that such homeostatic mechanism is disturbed by the presence of mutated APP and/or PS1, and may be relevant in Alzheimer's disease, as suggested by the generalized observation that DHA contents are reduced in the hippocampus of AD brains.

### Conflict of interest statement

The authors declare that the research was conducted in the absence of any commercial or financial relationships that could be construed as a potential conflict of interest.
